# Prevalence of BRCA1 in a hospital-based population of Dutch breast cancer patients

**DOI:** 10.1054/bjoc.2000.1331

**Published:** 2000-08-17

**Authors:** H Papelard, G H de Bock, R van Eijk, T P M Vliet Vlieland, C J Cornelisse, P Devilee, R A E M Tollenaar

**Affiliations:** Department of Medical Decision Making, Department of Surgery, Department of Pathology, Department of Human and Clinical Genetics, Leiden University Medical Center, The Netherlands

**Keywords:** BRCA1 prevalence, general breast cancer population, Dutch founder mutations

## Abstract

The prevalence of disease-related BRCA1 mutations was investigated in 642 Dutch breast cancer patients not selected for family history or age at diagnosis. They were tested for germline mutations in the BRCA1 gene using an assay which detects small deletions and insertions (DSDI), as well as the two major genomic founder deletions present in the Dutch population. Data on family history and bilateral breast cancer were obtained retrospectively. Ten protein truncating mutations were detected and one in-frame deletion with an unknown relation to disease risk. Four patients carried the Dutch founder deletion of exon 22. Based on these results the estimated prevalence of breast cancer in the general population in the Netherlands attributable to BRCA1 mutations is 2.1%. Under 40 years-of-age and under 50 years-of-age this prevalence is 9.5% and 6.4%, respectively. All mutation carriers were under 50 years-of-age at diagnosis of the first breast cancer, and five did not have any relative with breast cancer. The proportions of bilateral breast cancer in the mutation carriers and non-carriers did not differ from each other. These data indicate that in the general Dutch breast cancer population the great majority of BRCA1 mutations will be found in women diagnosed under 50 years-of-age. © 2000 Cancer Research Campaign


British Journal of Cancer (2000) 83(6), 719–724
© 2000 Cancer Research Campaign
doi: 10.1054/ bjoc.2000.1331, available online at http://www.idealibrary.com on IDEAL®

### Prevalence of BRCA1 in a hospital-based population of Dutch breast cancer patients

**H Papelard[1,2], GH de Bock[1], R van Eijk[3], TPM Vliet Vlieland[1], CJ Cornelisse[3], P Devilee[3,4] and RAEM Tollenaar[2]**

[1]Department of Medical Decision Making; [2]Department of Surgery; [3]Department of Pathology; [4]Department of Human and Clinical Genetics; Leiden University Medical Center, The Netherlands

**Summary** The prevalence of disease-related BRCA1 mutations was investigated in 642 Dutch breast cancer patients not selected for family history or age at diagnosis. They were tested for germline mutations in the BRCA1 gene using an assay which detects small deletions and insertions (DSDI), as well as the two major genomic founder deletions present in the Dutch population. Data on family history and bilateral breast cancer were obtained retrospectively. Ten protein truncating mutations were detected and one in-frame deletion with an unknown relation to disease risk. Four patients carried the Dutch founder deletion of exon 22. Based on these results the estimated prevalence of breast cancer in the general population in the Netherlands attributable to BRCA1 mutations is 2.1%. Under 40 years-of-age and under 50 years-of-age this prevalence is 9.5% and 6.4%, respectively. All mutation carriers were under 50 years-of-age at diagnosis of the first breast cancer, and five did not have any relative with breast cancer. The proportions of bilateral breast cancer in the mutation carriers and non-carriers did not differ from each other. These data indicate that in the general Dutch breast cancer population the great majority of BRCA1 mutations will be found in women diagnosed under 50 years-of-age. © 2000 Cancer Research Campaign

**Keywords**: BRCA1 prevalence; general breast cancer population; Dutch founder mutations

Since the identification of BRCA1 (Miki et al, 1994) and BRCA2 (Wooster et al, 1995), presymptomatic DNA screening for disease-related BRCA1 and BRCA2 mutations has become possible. Now that more breast cancer patients seem to be aware of the existence of these tests, a greater demand for testing on germline BRCA1/2 mutations can be expected. Despite clinical benefits, testing all breast cancer patients for genetic predisposition is, however, still controversial. It is hampered by, among other things, an incomplete knowledge about the frequency of disease-related mutations among patients not selected on the basis of family history status.

In two populations of young breast cancer patients not selected on family history, prevalences of disease-related BRCA1 mutations of 7.5% and 3.8% respectively have been observed (Langston et al, 1996; Southey et al, 1999). In another breast cancer population, not selected for age at diagnosis or family history, the frequency of disease-related BRCA1 mutations was 3.3% (Newman et al, 1998). However, the prevalence of BRCA1/2 mutations may differ between populations, while certain mutations may predominantly be found in populations with a specific ethnic background. The frequencies of such founder mutations, like the 185delAG and the 5382insC in BRCA1 observed among Ashkenazi Jews, may be much higher when compared to non-founder mutations. Among unselected Ashkenazi breast cancer patients the observed prevalence of the two founder mutations together is 3.7–8.9% (Abeliovich et al, 1997; Fodor et al, 1998; Hartge et al, 1999).

*Received 21 September 1999*
*Revised 3 April 2000*
*Accepted 11 May 2000*

*Correspondence to:* H Papelard, Department of Medical Decision Making, Leiden University Medical Center K-6-R, PO Box 2300 RC Leiden, The Netherlands

Several mutations in BRCA1 have been identified as major founders in the Netherlands (Peelen et al, 1997; Petrij-Bosch et al, 1997). To gauge the contribution of BRCA1 mutations to the incidence of breast cancer in the general population, a hospital-based study was undertaken. Breast cancer patients diagnosed between 1984 and 1996 at the Leiden University Medical Center (LUMC) were tested for the presence of germline BRCA1 mutations. Several characteristics (age at diagnosis of the first breast cancer, family history of cancer and bilateral breast cancer) were collected, to assess their relation to BRCA1 mutation status in this particular patient population.

### MATERIALS AND METHODS

### Population

From January 1984 to November 1996 all primary invasive breast cancer patients who were surgically treated at the Leiden Academic Hospital (LUMC), regardless of age or family history, were asked to provide a blood sample for research purposes. The total number of patients treated at the LUMC for invasive breast carcinoma in this period was 1099. This has led to 642 (58%) patients from whom lymphocyte DNA was available. Two of these patients were male. A total of 457 patients, including six males, did not provide a blood sample because they were not asked, or declined, to participate. There was no statistically significant difference between the patients who did or who did not provide a blood sample with respect to mean age at diagnosis of their first breast cancer (57.5 years-of-age, 95%CI: 56.4–58.6 and 58.2 years-of-age, 95%CI 56.8–59.5, respectively). The range of ages at diagnosis was 27–91 years-of-age and 23–96 years-of-age, respectively, with a median of 58 years for both groups.

Data were obtained regarding the family history of cancer of first- and second-degree relatives. The following was assessed: number of relatives, number of relatives, alive and dead, number of relatives with cancer, all related to type of relationship to index. For the affected relatives the following was assessed: type of relationship with index, type of cancer and bilaterality, if there was any breast cancer. We did not confirm information by pathology or other evidence. As the medical record provided limited information on family history, the general practitioners of the patients alive on 1 June 1997 ($n$ = 427) were contacted. This resulted in the approval to send a questionnaire to 318 patients. Completed questionnaires were received from 269 patients (overall response rate of 63%). For the patients who had died before 1 June 1997 ($n$ = 215), information was collected from the medical records and additional information was retrieved from the general practitioner of the patients. For the patients who did not fill in the questionnaire but were alive on 1 June 1997 ($n$ = 158), the only source of information was the medical record.

Data on the prevalence of bilateral breast cancer (synchronic and metachronic) among the 642 tested patients, were collected from the tumour registry of the LUMC, assessed August 1997. The 642 patients of whom lymphocyte DNA was available were tested anonymously for mutations in BRCA1. The Committee of Medical Ethics of the LUMC approved the study.

### The Dutch BRCA1 mutation spectrum

A national survey in January 1999 among eight clinical genetic centres in the Netherlands resulted in a total of 370 independently identified breast cancer families with a known mutation in BRCA1. In general, counsellees are offered a DNA test when they have an *a priory* chance >10% of being a BRCA1 carrier (Couch et al, 1997; Peelen et al, 1997; Shattuck-Eidens et al, 1997). The DNA test consists of the PTT for exon 11 (Hogervorst et al, 1995), supplemented with fragment-analysis (by SSCP or DGGE) of the smaller exons (usually exons 2, 5, 16, 20), and specific PCRs for the junction fragments of the large genomic founder deletions among the Dutch (Petrij-Bosch et al, 1997). The resulting mutation spectrum presently consists of 69 different mutations, 10 of which comprise 68% of the total set. There are 295 frame-shifting, 49 nonsense, 29 splice-site and four unclassified variants in this set, but no missense mutations.

### DNA-isolation and genomic PCR

Genomic DNA was extracted from peripheral white blood cells with a salting-out method (Millar et al, 1998). All PCR reactions were carried out in 15 µl volume in multiwell plates, containing approximately 100 ng $\mu l^{-1}$ genomic DNA, 5–10 pmol of each primerset, 0.75 U AmpliTaq polymerase (Perkin Elmer/Cetus) and 1.5 µl × 10 RM buffer (500 mM KCl, 100 mM TRIS-HCl pH 8.4, 25 mM $MgCl_2$, 2 mg $ml^{-1}$ BSA, 2 mM dNTPs). After heating at 94°C for 5 min, the PCR consisted of 30 cycles (45 s at 94°C, 1 min at 58°C and 1.5 min at 72°C), followed by an additional step of 5 min at 72°C.

### Detection of small deletions and insertions (DSDI)

Twenty primer pairs were designed in the BRCA1 regions in such a way that the following mutations could be detected: 1129delA, 1135insA, 1191delC, 1240delC, 1406insA, 1411insT, 1438delT, 185delAG, 185insA, 2138delA, 2312del5, 2329delC, 2331del4, 2804delAA, 2809insA, 2845insA, 2846del4, 2883del4, 3109insAA, 3600del11, 3604delA, 3875del4, 3889delAG, 3938insG, 4010delTTC, 4184del4, 4284delAG, 4370delGT, 5256delG, 5382insC, 5389del7, 5448insC, del ex 13–16, IVS12–1643del3835, IVS21–36del510. Using the information available on the Dutch Mutation Database (see section Electronic database information) it can be estimated that aproximately 75% of all currently known Dutch and Belgian mutations, including the large genomic deletions (Peelen et al, 1997; Petrij-Bosch et al, 1997) are traced with DSDI. All forward primers were labeled with a Hex, Tet or 6Fam fluorescent label. To facilitate multiplexing a 20mer M13 tail (5′-GCG GTC CCA AAA GGG TCA GT) was attached to forward and reversed primers (Shuber et al, 1995). Three multiplex PCR reactions were developed to amplify all fragments (available upon request). Subsequently PCR samples from the three different reactions were pooled in a 1:1:3 ratio for Tet, 6Fam and Hex respectively. 1 µl was taken and 0.5 µl TAMRA-500, 0.5 µl Dextran-Blue and 3.5 µl formaline were added. After 3 min denaturation at 94°C 1 µl of the sample mixture was loaded into a 6% Longranger™ gel on an AB1377-XL™ automated sequencer. Electrophoresis was performed for 7 h at 1680 V, 51°C at filterset C. Gel analysis was performed with GeneScan Analysis® 3.1 software. In the parameter files the Smooth Options and the Slit Peak Correction were set to None. For the Size Calling Method the second-order-least-squares option was used. After gel analysis, sample data were transferred to Genotyper™ 2.0 program. Software was programmed to call for suspected mutations if two peaks were present in a selected window. Possible variants were reamplified as simplex PCR products. After confirmation, the potential variants were directly sequenced from both strands on an AB1377 automatic sequencer using the BigDye Terminator (Applied Biosystems of Perkin Elmer) chemistry and the same primers for amplification of the fragments.

### Statistical methods

Assuming that DSDI will detect ~75% of Dutch and Belgian mutations, the mutation frequency in the general population in the Netherlands was considered to be 1/0.75 times the detected frequency. For the proportion of cases that carry a germline BRCA1 mutation the 95% confidence interval was calculated using the exact method described by Martin and Altman (1989) (exact method available upon request). For the estimated proportion that carry a germline mutation in the general population, the upper and lower limit of the 95% confidence interval were considered to be 1/0.75 times the limits given by the confidence interval of the observed proportion.

### RESULTS

Ten out of 642 genotyped breast cancer patients (1.6%) (Figure 1) contained a protein-truncating BRCA1 mutation (Table 1). One of these mutations, 2845insA in exon 11, had never been reported before. The 510bp deletion of exon 22 (del510) was observed four times in this series, whereas the other observed Dutch founder mutations, the 3835bp deletion of exon 13 (del3835), 1411insT and 2804delAA, were each detected once. The two remaining

*© 2000 Cancer Research Campaign*

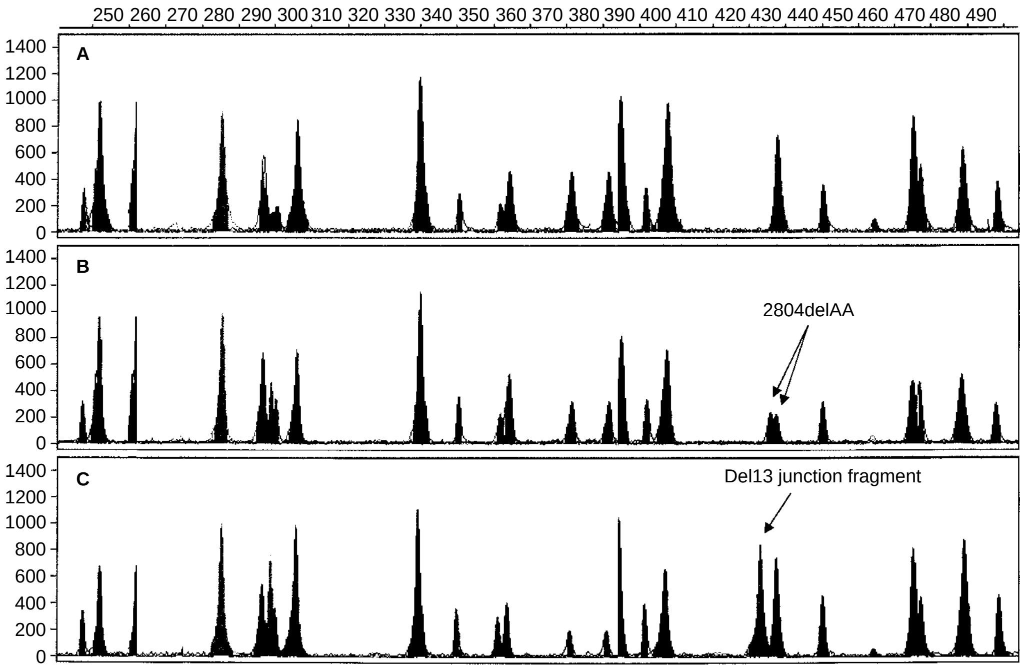

**Figure 1** DSDI pattern of a healthy control (A), of patient MC9 (B) and of patient MC7 (C)

**Table 1** Characteristics of mutation carriers and types of the observed BRCA1 mutations

| Identification code | Age at diagnosis (years) | Bilateral breast cancer (age at diagnosis) | Mutation | Exon | Type | Change | No. of citations in BIC[a]/DMD[b] |
|---|---|---|---|---|---|---|---|
| **disease-related** | | | | | | | |
| MC1 | 33 | no | del510 | 22 | Frameshift | stop 1805 | 35/57 |
| MC2 | 45 | no | del510 | 22 | Frameshift | stop 1805 | 35/57 |
| MC3 | 46 | no | del510 | 22 | Frameshift | stop 1805 | 35/57 |
| MC4 | 48 | no | del510 | 22 | Frameshift | stop 1805 | 35/57 |
| MC5 | 30 | yes(43) | 3875del4 | 11 | Frameshift | stop 1262 | 22/3 |
| MC6 | 34 | no | 5382insC | 20 | Frameshift | stop 1829 | 136/17 |
| MC7 | 35 | no | del3835 | 13 | Frameshift | stop 1398 | 22/30 |
| MC8 | 42 | no | 1411insT | 11 | Frameshift | stop 434 | 13/21 |
| MC9 | 45 | no | 2804delAA | 11 | Frameshift | stop 901 | 42/45 |
| MC10 | 45 | no | 2845insA | 11 | Frameshift | stop 916 | 0/0 |
| **relation with disease unknown** | | | | | | | |
| MC11 | 39 | no | 4010delTTC | 11 | In-frame deletion | deletion serine 1297 | 0/0 |

[a] Breast Cancer Information Core (assessed January 1999); [b] Dutch and Belgian Mutation Database (*n* = 370)

protein-truncating mutations were the 3875del4 and the Jewish founder 5382insC. Furthermore, one inframe deletion was detected, of which relation with disease-risk is unknown. All mutation carriers were females with a mean age at diagnosis of the first breast cancer of 40.3 years (95%CI 36.2–44.4) and a range of 30–48 years-of-age. Stratified by age group the highest prevalence (7.8%) was observed between 30 and 39 years-of-age at diagnosis (see Table 2). All other mutation carriers were observed among breast cancer patients of 40–49 years at diagnosis. In this age-group the prevalence of BRCA1 mutations was 3.9%. Adjusted for the 75% sensitivity of DSDI the prevalence under 40 years-of-age and under 50 years-of-age was estimated to be 9.5% and 6.4%, respectively. The prevalence of disease related BRCA1 mutations in the general population not selected for age at diagnosis was estimated to be 2.1% (see Table 2).

Complete data on the family history of cancer were available from the six mutation carriers (MC1–6) who filled in the questionnaire (Table 3). From the other four mutation carriers two (MC7, MC8) had died, and two (MC9 and MC10) did not return the questionnaire. From the patients who did not fill in the questionnaire the information regarding family history of cancer was mostly limited to breast cancer and only if the relative developed breast

© 2000 Cancer Research Campaign

**Table 2** Frequency of disease-related BRCA1 mutations by age-group

| Age-group | Number tested | Proportion of total (%) | Number positive | Proportion positive (% (95% CI)) | Adjusted cumulative proportion positive (%[a] (95%CI)) | Proportion predicted (%)[b] |
|---|---|---|---|---|---|---|
| <30 | 5 | 0.8 | 0 | 0 (0–52) | 0 (0–69) | 7.5 |
| 30–39 | 51 | 7.9 | 4 | 7.8 (2.2–18.9) | 9.5 (2.7–23.1) | 5.1 |
| 40–49 | 154 | 24.0 | 6 | 3.9 (1.5–8.2) | 6.4 (3.1–11.5) | 2.2 |
| 50–59 | 158 | 24.6 | 0 | 0 (0–2.3) | 3.6 (1.7–6.6) | 1.4 |
| 60–69 | 128 | 19.9 | 0 | 0 (0–2.8) | 2.7 (1.3–4.9) | 0.8 |
| 70+ | 146 | 22.7 | 0 | 0 (0–2.5) | 2.1 (1.1–3.9) | – |
| **total** | **642** | **100** | **10** | **1.6 (0.8–2.9)** | **2.1 (1.1–3.9)** | **1.70** |

[a] Assuming that DSDI will have missed 25% of the mutations; [b] Proportion predicted by Ford et al (1995)

**Table 3** Family history of cancer in the disease-related BRCA1 mutation carriers

| Identification code | Number of first-degree relatives with breast (br) or ovarian (ov) cancer[b] | Number of second-degree relatives with breast (br) or ovarian (ov) cancer[c] | Total number of first/ second-degree female relatives | Total Number of relatives with malignancies other than breast or ovarian cancer | Type of malignancy and relative[b] with this type of malignancy |
|---|---|---|---|---|---|
| MC1 | 0 | 2 ov (p) | 2/5 | 4 | lymphoma (Pu), throat or lung cancer (Pu), gastric cancer (Gf), leukaemia (Gf) |
| MC2 | 1 br (S) | 1 br (p) | 4/6 | 7 | cancer of intestine (B, Pu), lung carcinoma (F), leukaemia (2 Ma), Esophageal carcinoma (Pu) |
| MC3 | 0 | 2 br (p, m) | 2/11 | 2 | lung carcinoma (2 Pu) |
| MC4 | 0 | 0 | 1/6 | 9 | throat carcinoma (M), lung cancer (F, 3 Mu), cancer of intestine (Mu, Gmm), liver cancer (Pu, Gmp) |
| MC5 | 1 br (S), 1 ov (M) | 3 br (2m, p) | 2/7 | 1 | melanoma (Gfp) |
| MC6 | 1 br (S) | 0 | 4/3 | 2 | leukaemia (S), uterine cancer (M) |
| MC7[a] | 0 | 0 br, ? ov | unknown | 1 known | thyroid carcinoma (F) |
| MC8[a] | 0 | 0 br, ? ov | unknown | 1 known | melanoma (B) |
| MC9[a] | 0 br, ? ov | 0 br, ? ov | unknown | unknown | |
| MC10[a] | 0 br, ? ov | 0 br, ? ov | unknown | unknown | |

[a] Patient did not fill in the questionnaire; [b] F = Father, M = Mother, S = Sister, B = Brother, Pu/Pa = paternal uncle/aunt, Mu/Ma = maternal uncle/aunt, Gmp/Gfp = paternal grandmother/father, Gmm/Gfm = maternal grandmother/father; [c]p = paternal, m = maternal; ? ov = history of ovarian cancer is not known cancer before our proband was diagnosed. Five out of 10 mutation carriers had a first-or second-degree relative with breast cancer. Even when the mutation carriers who did not fill in the questionnaire are excluded, the proportion of mutation carriers without a first-degree relative with breast or ovarian cancer is three out of six. The proportion of first-degree or second-degree relatives with breast and/or ovarian cancer, however, is one out of six. Bilateral breast cancer was recorded in 61 out of 631 mutation-negative patients (9.7%, 95%CI 7.5–12.2%) and in one out of 10 mutation-positive patients (10%, 95%CI 2.5–44.5%).

### DISCUSSION

A hospital-based population of 642 breast cancer patients, unselected for family history or age at diagnosis, was screened for disease-related BRCA1 mutations. This study population appears to be a good representation of the general breast cancer population in the Netherlands. Although a slight bias towards younger age cases was seen in our series compared to the Dutch breast cancer population in general (Coebergh et al, 1995), the difference was so subtle that it cannot plausibly have made a dramatic impact on our prevalence estimates. The likelihood of selecting patients with a positive family history of breast or ovarian cancer is probably small, since most of the patients were included before 1995, i.e. before public awareness on hereditary cancer increased due to the identification of BRCA1. Furthermore, we think that medical awareness on hereditary cancer was also limited since we did not find much information regarding family history in the medical record.

Ten disease-related BRCA1 mutations were detected in this study population (1.6%), which is probably an underestimate

 *© 2000 Cancer Research Campaign*

considering that our screening-assay is not 100% sensitive. This prevalence is in accordance with previous estimations by the Breast Cancer Linkage Consortium (BCLC) (Ford et al, 1995) (Table 1), but slightly lower than the three carriers found among 120 white breast cancer patients from a US North Carolina population (Newman et al, 1998). Thus, despite strong founder-effects of several BRCA1 mutations among the Dutch (Peelen et al, 1997), the overall population frequency of BRCA1 does not seem to be higher than predicted by the BCLC. This is in sharp contrast with the Ashkenazi Jewish population, where two BRCA1 founder-mutations occur at a frequency eight times that of other populations. Our observed prevalence does not substantially differ from predictions by the BCLC, which suggests that the overall BRCA1 penetrance will also come out as estimated by the BCLC, i.e. about 50% before age 50 (Easton et al, 1995). However, an extrapolation to the general breast cancer population in the Netherlands should be performed with caution, firstly because it assumes that our assay will detect 75% of all mutations prevalent among the Dutch. Even though over 1500 families have been screened in eight Cancer Family Clinics, this screening does not entail the entire BRCA1 coding region and therefore the currently known 'Dutch mutation spectrum' might still be incomplete. In addition, this spectrum could be different from the one present among the general breast cancer population. Secondly, our study population is clinic-based and may therefore differ from the general breast cancer population in the Netherlands, even though this is not supported by our clinical data.

All BRCA1 mutation carriers, or 10 out of 210 (5%), were under 50 years-of-age at diagnosis of their first breast cancer. We found no carrier that had been diagnosed under 30 years-of-age. Previous population-based studies also found small proportions of mutation carriers among patients under 30 (Langston et al, 1996; Newman et al, 1998; Southey et al, 1999), with one exception reporting 11.4% (Malone et al, 1998). Based on linkage data from high-risk families, the BCLC estimated that 7.5% of the patients in this age-group would be carrying a BRCA1 mutation (Table 1). Families with multiple cases of breast cancer in different generations were needed for this type of analysis and selection for extremely young ages at diagnosis could have occurred. Nonetheless, the small number of patients under 30 in our series ($n$ = 5), and in other studies (Langston et al, 1996; Newman et al, 1998; Southey et al, 1999), results in wide confidence intervals. Further research will therefore be required to establish the prevalence of BRCA1 in this particular group of patients.

An increased risk of having a BRCA1 or BRCA2 mutation was observed among Dutch families with a small number of female relatives if there is a relative with bilateral breast cancer present (Ligtenberg et al, 1999). The results of our study, however, suggest that bilateral breast cancer (as an isolated parameter) in the patient herself is not indicative for BRCA1 mutation status, since the proportion of patients with bilateral breast cancer in our study was the same in carriers and non-carriers.

The number of carrier patients in our study without a first-degree relative with breast or ovarian cancer is striking. This is probably due to a stochastic deficit of first-degree females at risk (Table 3). When both first- and second-degree female relatives are included, the proportion of carrier patients with a positive family history is much higher. The observation that a number of mutation carriers do not have a relative with breast or ovarian cancer is nevertheless in accordance with previous studies by others (FitzGerald et al, 1996; Tonin et al, 1996; Abeliovich et al, 1997; Hartge et al, 1999). It could mean that some mutations confer a lower penetrance than previously estimated, as has been reported for the 185delAG and the 5382insC mutations in the Ashkenazi Jewish population (Struewing et al, 1997; Fodor et al, 1998). A candidate for such a reduced penetrance mutation in the Netherlands is the del510, since this mutation was observed four times in our series, whereas 1.5 were expected on the basis of its prevalence in high-risk families (Table 1). In contrast, the Dutch founder-mutations del3835, 1411insT and 2804delAA were identified in the proportions anticipated on this basis. As the number of affected relatives among the four carriers of the del510 mutation shows a wide variation, it seems likely that this mutation causes heterogeneous cancer risks in different families through genetic and environmental modifying effects (Devilee, 1999).

In conclusion, our results indicate that about 5% of the Dutch breast cancer patients diagnosed under the age of 50 can be attributed to BRCA1. Furthermore, a substantial proportion of mutation carriers did not have a first-degree relative with breast cancer. This suggests that the overall BRCA1 penetrance in the Dutch population will likely come out in the same order of magnitude as estimated previously by the BCLC, although some evidence for risk heterogeneity was also obtained. Further investigation is needed to establish the full mutation spectrum and associated cancer risks in each category of patients.

### ACKNOWLEDGEMENT

We are grateful to F Hogervorst from the Dutch National Cancer Institute for collecting and making available BRCA1 mutation data from all Clinical Genetic Centers in the Netherlands and to the many breast cancer patients and their general practitioner who participated in the research. This work was supported by a grant from the Leiden University Medical Center, Leiden, the Netherlands.

### Electronic database information

URLs for data in this article are as follows: Breast Cancer Information Core, http://www.nhgri.nhi.gov/intramural_research/lab_transfer/bic/ Dutch Mutation Database, http://ruly70.medfac.leidenuniv.nl/~devilee/lab/blnl5.htm

### REFERENCES

Abeliovich D, Kaduri L, Lerer I, Weinberg N, Amir G, Sagi M, Zlotogora J, Heching N and Peretz T (1997) The founder mutations 185delAG and 5382insC in BRCA1 and 6174delT in BRCA2 appear in 60% of ovarian cancer and 30% of early-onset breast cancer patients among Ashkenazi women. *Am J Hum Genet* **60**: 505–514

Coebergh JW, van der Heijden LH and Janssen-Heijnen ML (1995) Cancer incidence and survival. *Comprehensive Cancer Center South*: 54–55

Couch FJ, DeShano ML, Blackwood MA, Calzone K, Stopfer J, Campeau L, Ganguly A, Rebbeck T and Weber BL (1997) BRCA1 mutations in women attending clinics that evaluate the risk of breast cancer. *N Engl J Med* **336**: 1409–1415

Devilee P (1999) BRCA1 and BRCA2 testing: Weighting the demand against the benefits. *Am J Hum Genet* **64**: 943–948

Easton DF, Ford D and Bishop DT (1995) Breast and ovarian cancer incidence in BRCA1-mutation carriers. Breast Cancer Linkage Consortium. *Am J Hum Genet* **56**: 265–271

FitzGerald MG, MacDonald DJ, Krainer M, Hoover I, O'Neil E, Unsal H, Silva AS, Finkelstein DM, Beer RP, Englert C, Sgroi DC, Smith BL, Younger JW, Garber

© 2000 Cancer Research Campaign

JE, Duda RB, Mayzel KA, Isselbacher KJ, Friend SH and Haber DA (1996) Germ-line BRCA1 mutations in Jewish and non-Jewish women with early-onset breast cancer. *N Engl J Med* **334**: 143–149

Fodor FH, Weston A, Bleiweiss IJ, McCurdy LD, Walsh MM, Tartter PI, Brower ST and Eng CM (1998) Frequency and carrier risk associated with common BRCA1 and BRCA2 mutations in Ashkenazi Jewish breast cancer patients. *Am J Hum Genet* **63**: 45–51

Ford D, Easton DF and Peto J (1995) Estimates of the gene frequency of BRCA1 and its contribution to breast and ovarian cancer incidence. *Am J Hum Genet* **57**: 1457–1462

Hartge P, Struewing J, Wacholder S, Brody LC and Tucker MA (1999) The prevalence of common BRCA1 and BRCA2 mutations among Ashkenazi Jews. *Am J Hum Genet* **64**: 963–970

Hogervorst F, Cornelis RS, Bout M, van Vliet M, Oosterwijk JC, Olmer R and Bakker B (1995) Rapid detection of BRCA1 mutations by the protein truncation test. *Nat Genet* **10**: 208–212

Langston AA, Malone KE, Thompson JD, Daling JR and Ostrander EA (1996) BRCA1 mutations in a population-based sample of young women with breast cancer. *N Engl J Med* **334**: 137–142

Ligtenberg MJ, Hogervorst FB, Willems HW, Arts PJ, Brink G, Hageman S, Bosgoed EA, Van-der-Looij E, Rookus MA, Devilee P, Vos EM, Wigbout G, Struycken PM, Menko FH, Rutgers EJ, Hoefsloot EH, Mariman EC, Brunner HG and van't-Veer LJ (1999) Characteristics of small breast and/or ovarian cancer families with germline mutations in BRCA1 and BRCA2. *Br J Cancer* **79**: 1475–1478

Malone KE, Daling JR, Thompson JD, O'Brien CA, Francisco LV and Ostrander EA (1998) BRCA1 mutations and breast cancer in the general population: analyses in women before age 35 years and in women before age 45 years with first-degree family history. *JAMA* **279**: 922–929

Martin JG and Altman GA (1989) Statistics with confidence – confidence intervals and statistical guidelines. British Medical Journal Publications: London

Miki Y, Swensen J, Shattuck-Eidens D, Futreal PA, Harshman K, Tavtigian S, Liu Q, Cochran C, Bennett LM, Ding W, Bell R, Rosenthal J, Hussey C, Tran T, McClure M, Frye C, Hattier T, Phelps R, Haugen-Strano A, Katcher H, Yakumo K, Gholami Z, Shaffer D, Stone S, Bayer S, Wray C, Bogden R, Dayananth P, Ward J, Tonin P, Narod S, Bristow PK, Norris FH, Helvering L, Morrison P, Rosteck P, Lai M, Barret JC, Lewis C, Neuhausen S, Cannon-Albright L, Goldgar D, Wiseman R, Kamb A and Skolnick MH (1994) A strong candidate for the breast and ovarian cancer susceptibility gene BRCA1. *Science* **266**: 66–71

Millar SA, Dykes DD and Polesky HF (1998) A simple salting out procedure for extracting DNA from human nucleated cells. *Nucleic Acids Res* **16**: 1215

Newman B, Mu H, Butler LM, Millikan RC, Moorman PG and King MC (1998) Frequency of breast cancer attributable to BRCA1 in a population-based series of American women. *JAMA* **279**: 915–921

Peelen T, van Vliet M, Petrij-Bosch A, Mieremet R, Szabo C, van-den-Ouweland AM, Hogervorst F, Brohet R, Ligtenberg MJ, Teugels E, van-der-Luijt R, van-der-Hout AH, Gille JJ, Pals G, Jedema I, Olmer R, van L, I, Newman B, Plandsoen M, van-der-Est M, Brink G, Hageman S, Arts PJ, Bakker MM and Devilee P (1997) A high proportion of novel mutations in BRCA1 with strong founder effects among Dutch and Belgian hereditary breast and ovarian cancer families. *Am J Hum Genet* **60**: 1041–1049

Petrij-Bosch A, Peelen T, van-Vliet M, van-Eijk R, Olmer R, Drusedau M, Hogervorst FB, Hageman S, Arts PJ, Ligtenberg MJ, Meijers HH, Klijn JG, Vasen HF, Cornelisse CJ, van't-Veer LJ, Bakker E, van-Ommen GJ and Devilee P (1997) BRCA1 genomic deletions are major founder mutations in Dutch breast cancer patients. *Nat Genet* **17**: 341–345

Shattuck-Eidens D, Oliphant A, McClure M, McBride C, Gupte J, Rubano T, Pruss D, Tavtigian SV, Teng DH, Adey N, Staebell M, Gumpper K, Lundstrom R, Hulick M, Kelly M, Holmen J, Lingenfelter B, Manley S, Fujimura F, Luce M, Ward B, Cannon AL, Steele L, Offit K and Thomas A (1997) BRCA1 sequence analysis in women at high risk for susceptibility mutations. Risk factor analysis and implications for genetic testing. *JAMA* **278**: 1242–1250

Shuber AP, Grondin VJ and Klinger KW (1995) A simplified procedure for developing multiplex PCRs. *Genome Res* **5**: 488–493

Southey MC, Tesoriero A, Andersen CR, Jennings KM, Brown SM, Dite G, Jenkins MA, Osborne RH, Maskiell JA, Porter L, Giles GG, McCredie MR, Hopper JL and Venter DJ (1999) BRCA1 mutations and other sequence variants in a population-based sample of Australian women with breast cancer. *Br J Cancer* **79**: 34–39

Struewing JP, Hartge P, Wacholder S, Baker SM, Berlin M, McAdams M, Timmerman MM, Brody LC and Tucker MA (1997) The risk of cancer associated with specific mutations of BRCA1 and BRCA2 among Ashkenazi Jews. *N Engl J Med* **336**: 1401–1408

Tonin P, Weber B, Offit K, Couch F, Rebbeck TR, Neuhausen S, Godwin AK, Daly M, Wagner CJ, Berman D, Grana G, Fox E, Kane MF, Kolodner RD, Krainer M, Haber DA, Struewing JP, Warner E, Rosen B, Lerman C, Peshkin B, Norton L, Serova O, Foulkes WD, Lynch HT, Lenoir GM, Narod SA and Garber JE (1996) Frequency of recurrent BRCA1 and BRCA2 mutations in Ashkenazi Jewish breast cancer families. *Nat Med* **2**: 1179–1183

Wooster R, Bignell G, Lancaster J, Swift S, Seal S, Mangion J, Collins N, Gregory S, Gumbs C and Micklem G (1995) Identification of the breast cancer susceptibility gene BRCA2. *Nature* **378**: 789–792

 *© 2000 Cancer Research Campaign*
